# Alzheimer's disease: Still in need of a cure!

**DOI:** 10.1016/j.eclinm.2021.101146

**Published:** 2021-09-24

**Authors:** EClinicalMedicine

September 2021 marks the 10 year anniversary of the World Alzheimer's Month campaign intended to raise awareness of the disease and its’ challenges. The campaign was founded as a truly global effort to fight misinformation and stigma surrounding Alzheimer's disease (AD). World Alzheimer's Day occurs every year on Sept 21st. This year's theme for World Alzheimer's Month is “Know dementia, know Alzheimer's” with a special focus on the diagnosis and early warning signs of AD.

AD is the most common form of dementia and it affects part of the brain controlling memory, cognition, language, and behavioural functions. Worldwide, around 50 million people are currently living with AD or other types of dementia. In 2020, as many as almost 6 million people in the USA alone were diagnosed with AD which is expected to nearly triple by 2060. First symptoms generally appear after the age of 60 years and usually increase with age. There are certain risk factors that are known to contribute to the development of AD. Age is probably the greatest known risk factor, whereas family history and genetics are other strong predictors. Although such risk factors cannot be changed, some research highlights that strategies for overall healthy aging, such as exercise, staying mentally and socially active, and a balanced diet, might be beneficial in the prevention of AD.

The most common and earliest symptoms for the onset of AD are difficulties with remembering current events and new information. More severe symptoms include confusion, disorientation, mood and behaviour changes, and difficulties in speaking, walking, and swallowing. The actual cause of AD is still under active debate in the field, but it is believed that microscopic changes in the brain even long before any symptom onset play a crucial role. There are currently two main scientific theories proposed that are believed to contribute to the disease. Both involve the accumulation of proteins in the brain, tau or β amyloid, which are suspected to cause neurotoxicity that could affect the function of brain cells causing the known symptoms of AD. Autopsy findings confirmed that patients who died with AD show accumulation and aggregates of such plagues or tangles in the brain. However, it is important to point out that such aggregates are also present in the brains of older deceased people who had not manifested any AD symptoms during their lifetime.

Since the actual cause of AD remains mostly unknown, diagnosis, and early detection is still a main focus of research. Neuroimaging is among the most promising areas of research. Active research is also underway to establish biomarkers such as soluble β amyloid or tau concentrations in the CSF, or the use of linguistic changes in order to be able to diagnose AD earlier. One of the biggest challenges for a reliable diagnosis is the fact that there seems to be huge discrepancy between diagnosed and actual clinical manifestations of AD. Using the National Institute on Aging–Alzheimer's Association framework for AD diagnosis, which includes molecular imaging based on β amyloid plaques or tau tangles but not clinical symptoms, shows that patients tend to get diagnosed 3 times more often with AD but only a third will ever show any symptoms. This discrepancy sheds doubt on the involvement of β amyloid plaques or tau tangles in the clinical manifestation, cause, and progression of AD. This also highlights the need for better and reliable diagnostic tools to be able to identify patients with AD before symptoms are present.

Since the actual cause of the disease is still unknown, there are also no viable treatment options available in the clinic. Currently the medical management of AD aims to improve the quality of life of the patients, including maintaining brain health and managing behavioural symptoms in the hope of delaying symptoms of the disease. In June, the drug Aducanumab by Biogen received accelerated approval by the US Food and Drug Administration, which caused huge uproar in the field since all clinical trials surrounding the drug showed no clinical benefit in reducing clinical decline in patients with AD. The only effect which was observed showed a dose and time dependent reduction in β amyloid plaques by the drug. Based on this finding the drug was approved even though the involvement of β amyloid plaques in the disease is highly controversial but what is maybe even more stunning without showing any clinical benefit in relieving symptoms nor in slowing the decline. Of course, this approval has significant financial implications since the drug would be available now for around 5.3 million people 65 years and older in the USA at an annual cost of $56 000—which is substantially higher compared with the estimated cost-effectiveness of $8300. The lack of clinical benefits and the discrepancy in the cost estimates led to the call of an independent investigation into the approval process by the House Committee on Oversight and Reform and the Committee on Energy and Commerce that is currently underway.

With this year's World Alzheimer's Day focus on the need for diagnosis, more research will hopefully lead to novel developments in this area. Overall, it becomes evident that AD remains a global health challenge with no reliable options for diagnosis and treatment leaving families, patients, and clinicians in urgent need of medical support and guidance.

